# Evolution of Tactics in Professional Soccer: An Analysis of Team Formations from 2012 to 2021 in the Spanish LaLiga

**DOI:** 10.5114/jhk/167468

**Published:** 2023-07-15

**Authors:** Joaquín González-Rodenas, Víctor Moreno-Pérez, Roberto López-Del Campo, Ricardo Resta, Juan Del Coso

**Affiliations:** 1 Centre for Sport Studies, Rey Juan Carlos University, Fuenlabrada, Madrid, Spain.; 2 Sports Research Center, Miguel Hernandez University of Elche, Alicante, Spain.; 3 Department of Competitions and Mediacoach, LaLiga, Madrid, Spain.

**Keywords:** football, game style, elite athlete, performance, team sport

## Abstract

The aim of this investigation was to explore the evolution of team formations (TFs) in a top national professional soccer league. A comparative analysis of a total of 3420 matches was performed in 32 professional soccer teams that competed from the 2012/13 to the 2020/21 season in the Spanish LaLiga. The frequency distribution of TFs across seasons presented a statistically significant change (p < 0.001). A 4.2-3.1 TF was most common from 2012/13 to 2016/17, while a 4.4.2 TF was most used from 2017/18 to 2020/2021. TFs such as 4.3.3 and 4.5.1 showed a relatively stable trend over the seasons. Overall, there was a reduction of TFs with 5 midfielders and an increase in the frequency of 2-forward formations (p < 0.001). When the ranking of teams was considered, a 4.3.3 TF was implemented with higher frequency by the Champion and teams that qualified for the Champions League (p < 0.05). However, a 4.2-3.1 TF was most frequent for teams qualified for the UEFA Europa League or teams that got relegated to an inferior category (p < 0.05). These findings highlight that teams competing in LaLiga evolved from the implementation of very few TFs led by the 4.2-3.1 TF, towards a more diverse scenario with a greater variety of tactical positional structures.

## Introduction

One of the key decisions of soccer coaches is to determine the tactical team formation (TF) of their team as a part of a whole match strategy to beat the opposing team (Mesoudi, 2020). The TF creates a collective organization that defines the spatial arrangement of players by grouping them usually into four tactical lines (goalkeeper, defenders, midfielders, and attackers) (Konefal et al., 2019).

Although the selection of a specific TF does not implicate to implement a determined style of play, it is a key aspect for any coach because it allows to distribute players in field positions where they can maximize their individual performance. In this sense, the objective of each TF is to create synergies and positive interactions between players. In fact, the selection of a specific TF represents a starting point from where players can move and interact with each other depending on the style of play adopted by the coach and the behavior of the opposing team. For this reason, the match-to-match selection of the TF plays a critical role in professional soccer. Additionally, the identification of the TFs is one of the first things that soccer analysts evaluate in their assessment of performance during matches (Low et al., 2022).

There has been a constant evolution of TFs throughout the history of soccer. While in the early years of the twentieth century teams presented formations with a high quantity of players in advanced lines (i.e., 2.3.5), the organization of players has notably changed to create more balanced structures between the offensive and defensive lines (i.e., 4.3.3, 4.4.2). In modern soccer, the most common TFs include the highest number of players in defensive and midfielders’ lines, while the 4.2-3.1 formation has been one of the most recurrent TFs in the last 10 years (Eliakim et al., 2022).

The evolution of soccer, which is now a more intense game with a higher number of matches per year, seems to have given place to the use of a greater number of TFs. For instance, during the 2018 Men´s World Cup, the 4.2-3.1 was the most predominant TF, followed by the 4.4.2, 4.3.3, 3.5.2, 4.5.1, 5.3.2 and 5.4.1 (Guedea-Delgado et al., 2019). In the top five European leagues, the 4.2-3.1, 4.4.2 and 4.3.3 are the most recurrent TFs, although it seems that there has been a clear reduction in the frequency of the 4.2-3.1, towards the use of the 4.4.2 formation. This is likely related to the fact that current teams perform an increased number of passes per match, especially short passes, and teams are now more focused on controlling the match and creating offensive space by increasing the passing frequency and accuracy (Yi et al., 2020). Particularly, the evolution of the Spanish *LaLiga* in the last years has shown that central backs have increased the number of passes, short passes, and long passes throughout the last eight seasons, as well as players made fewer tackles, clearances, but more interceptions and more high intensity efforts (Lago-Peñas et al., 2022). Besides, the tactical evolution for the future game predicts models of high intensity, pressing, counter pressing and counterattacking (Nassis et al., 2020).

Within this change of game demands, analysis of how professional soccer teams use TFs and their tactical effects is key to understand the evolution of professional soccer. In fact, recent studies have observed that physical demands and technical-tactical actions performed by professional soccer players highly depend on the TF used (Aquino et al., 2020; Arjol-Serrano et al., 2021; Bradley et al., 2011; Modric et al., 2020). For example, the study of Tierney et al. (2016) reported that the 3.5.2 TF elicited higher total distance, high-speed running and high metabolic load distance than 4.3.3, 4.4.2, 3.4.3 and 4.2-3.1 formations. Also, that study found that the 4.2-3.1 formation elicited the highest physical load in terms of accelerations and decelerations.

Despite the demonstrated technical-tactical and physical relevance of TFs (Memmert et al., 2019), very few studies have analyzed their evolution and use in professional soccer. Therefore, the aim of this investigation was to explore the evolution of TFs from the 2012/13 to the 2020/21 season of the Spanish *LaLiga*.

## Methods

### Sample

The sample included 32 professional soccer teams that competed in the first division of the Spanish *LaLiga* across 9 consecutive seasons (2012/13–2020/21). From this sample, only 9 teams competed in *LaLiga* in all the nine seasons under investigation. The remaining 21 teams competed in a range of 1 to 8 seasons because they got relegated to the second division of the professional Spanish league. During the period of analysis, a total of 3420 matches were played and hence, we obtained data from 6840 initial TFs. Data were collected from *LaLiga*, which authorized the analysis of variables included in this investigation and the publication of results with a scientific objective. In accordance with the ethical guidelines of *LaLiga*, this investigation does not include information that identifies particular players.

### Procedures

This investigation is a descriptive analysis to determine how TFs have evolved in professional Spanish soccer in the last seasons.

The design of the analysis of TFs included three main perspectives. Firstly, our study evaluated the evolution of the use of different TFs from the 2012/2013 to the 2020/2021 season, including the evolution of the number of defenders, midfielders and forwards in each tactical line of the teams. Secondly, our analysis compared the use of different TFs according to the team ranking and their association with the number of points obtained at the end of the season. For this purpose, teams were divided into five groups depending on their final ranking position (Champion = 1^st^ position; Champions League = 2^nd^ to 4^th^ positions; Europa League = 5^th^ and 6^th^ positions; middle teams = 7^th^ to 17^th^ position, and relegated teams = 18^th^ to 20^th^ positions). Finally, an evaluation of TFs used by specific teams that have stayed in the Spanish first division during the nine seasons evaluated was performed.

In this regard, all TFs were recorded by Mediacoach®, a multicamera tracking system that can accurately assess match statistics and player’s running distances during match play. In addition to match statistics and running patterns, Mediacoach® collects data on TFs using information from OPTA Sportsdata.

To determine the use of TFs during matches, two specialized and experienced operators recorded the position and movements of each team during the first 10 minutes of the match. These operators categorized the TF selected by each team’s manager considering the position of players during both the attacking and defending moments. Besides this, the categorization of TFs was supervised after the match by a third analyst who resolved disagreements (if any) between the two initial operators. Finally, it is important to note that all TFs excluded the goalkeeper from the analysis.

The operators had the option to select up to 21 different TFs. However, for the sake of readability, we grouped these 21 TFs into a simpler cluster of eight main TFs (Table 1). To make this classification, we used the number of players included in each of the lines that habitually conformed to the tactical structures of the teams: defenders, midfielders, and forwards. For example, the 4.1-4.1 formation was formed by three main lines, but in the midfielders’ line, there was one player further back than the other four midfielders, what is considered a variant of the 4.5.1 TF. We included all the variants within the same TF in a single group, except for the 4.2-3.1. For this specific case, the 4.2-3-1 can be considered a variant of the 4.5.1. However, it is crucial for the study to consider that the use of the 4.2-3.1 formation is more frequent than the 4.5.1 itself, as well as this TF is one of the most used in the five top European leagues (Mesoudi, 2020).

**Table 1:** Frequencies of initial team formations used by teams and their possible variants.

Team formation	n	Variants
**3.5.2**	232	3.1-4.2 / 3.5.1-1 / 3.2-3.2
**3.4.3**	190	3.4.2-1 / 3.4.1-2 / 3.1-2-1.3
**4.3.3**	1135	-
**4.5.1**	368	4.1-4.1 / 4.3-2.1
**4.2-3.1**	2663	-
**4.4.2**	2006	4.2-2.2 / 4.3-1.2 / 4.4.1-1, 4.1-3.2 / 4.1-2-1.2
**5.3.2**	155	-
**5.4.1**	91	-
**Total**	6840	

The number separated by a dot indicates different lines. The number separated by a hyphen represents a variant in the position of the players within the same line.

### Statistical Analysis

This study is a descriptive and comparative analysis. Statistical analyses were carried out using software IBM SPSS Statistics Version 27.0 (IBM Corp., Armonk, NY, United States). Descriptive statistics (frequencies) were calculated for the distribution of each TF within each season or within each group. A chi-square (χ2) test was used to compare the frequencies of TFs between seasons and among groups. To determine which TF was associated with an unexpected distribution, standardised residuals were calculated based on the difference between the observed and the expected values. Briefly, within a season/group, a TF was considered to have a statistically different distribution from the expected value when its distribution was > or < the critical Z-score value (*i.e.*, 1.96). The significance level was set at *p* < 0.050.

Also, a correlation analysis was performed to assess the association between the use of TFs and the final ranking. Initially, we checked the normality of each variable with the Kolmogorov-Smirnov test. None of the variables presented a normal distribution and thus, non-parametric statistics were employed. We calculated Spearman’s correlation coefficients (ρ) to assess the association between the frequency of each TF within each team and the number of points obtained at the end of the season.

## Results

**Figure 1:**
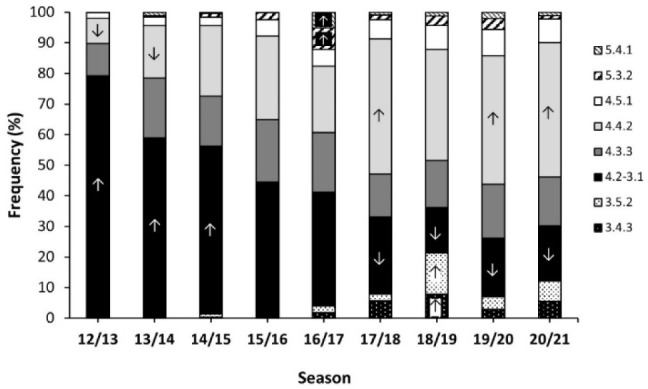
Distribution of teams’ formations at the beginning of the match in LaLiga from 2012/13 to 2020/21. Data are frequencies for each tactic formation within the same season. ↑ indicates that the selection of the tactic formation was higher than expected in that season at p < 0.05. ↓ indicates that the selection of the tactic formation was lower than expected in that season at p < 0.05.

Figure 1 depicts the distribution in the use of each TF across all the seasons under investigation. The frequency distribution of TFs across seasons presented a statistically significant change (*p* < 0.001). The 4.2-3.1 was the most common TF from 2012/13 to 2016/17, while the 4.4.2 TF was most common from 2017/18 to 2020/21. In fact, the use of the 4.2-3.1 TF was higher from 2012/13 to 2014/15 and then, it was lower in the last four seasons (all *p* < 0.050). Conversely, the use of the 4.4.2 TF was lower than expected in 2012/13 and 2013/14, seasons while it was higher than expected in 2017/18, 2019/20 and 2020/21 (all *p* < 0.050). Both the 5.3.2 and 5.4.1 TFs had higher frequencies than expected only in the 2016/17 season (*p* < 0.050), while 3.4.3 and 3.5.2 TFs were more prevalent only in the 2018/19 season (*p* < 0.050).

**Figure 2:**
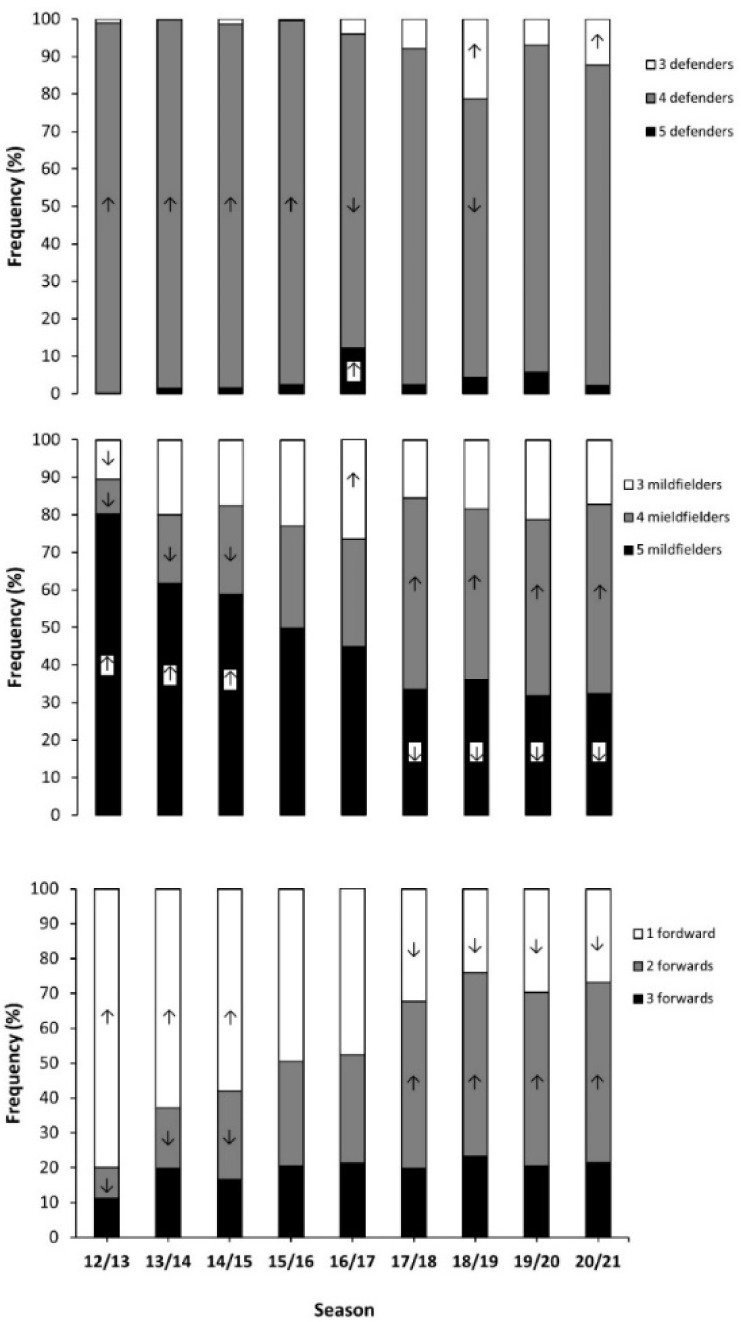
Distribution of teams’ formations at the beginning of the match in LaLiga according to the number of players in each line. Data are frequencies of team formations depending on the number of players per line within the same season. ↑ indicates that the selection of the number of players in each line was higher than expected in that season at p < 0.05. ↓ indicates that the selection of the number of players in each line was lower than expected in that season at p < 0.05.

Figure 2 presents a further analysis of the TF used across the seasons, but by merging formations with the same number of defenders, midfielders, and forwards, respectively. In all lines, there has been a change in the frequency of TFs across the seasons under investigation (all *p* < 0.001). TFs with four defenders were most common in all seasons under investigation, although a slight change was found since 2015/16. In the last four seasons, the use of TFs with three defenders was higher than expected, especially in 2018/19 and 2020/21 seasons (*p* < 0.050).

Regarding midfielders, the use of five midfielders was higher than expected from the 2012/13 to the 2014/15 season, although it decreased in the last four seasons (all *p* < 0.050). On the other hand, the use of four midfielders was lower from 2012/13 to 2014/2015, and it was higher in the last four seasons (all *p* < 0.050). Concerning forwards, the implementation of TFs with one forward was more prevalent from the 2012/13 to the 2014/15 season, although it had a descending trend in the last four seasons. On the contrary, TFs with two forwards presented an increasing trend in the last seasons (all *p* < 0.050).

**Figure 3:**
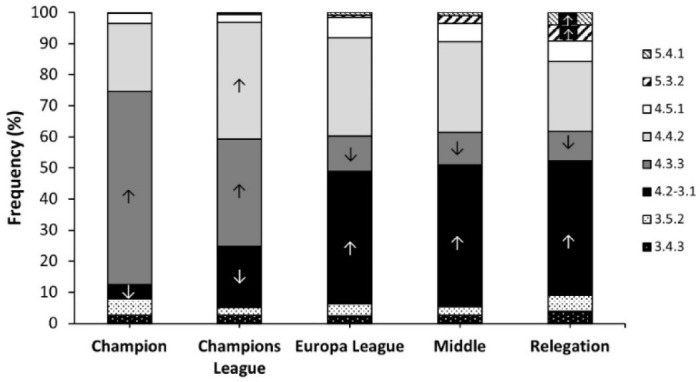
Distribution of teams’ formations at the beginning of the match according to the team’s ranking at the end of the season. *Data are frequencies for each tactic formation within the same group. ↑ indicates that the selection of the tactic formation was higher than expected in the group at p < 0.05. ↓ indicates that the selection of the tactic formation was lower than expected the group (p < 0.05)*.

Figure 3 shows the frequency of TFs according to the teams’ ranking at the end of the season (*p* < 0.001). The 4.3.3 was higher than its expected value in the team that won the league, while the use of the 4.2-3.1 was lower in this category (all *p* < 0.050). For the teams qualified for the UEFA Champions League, the use of the 4.3.3 TF was also higher, although the 4.4.2 was the most recurrent TF. For teams qualified for the UEFA Europa League, teams ranked in the middle of the standings and teams that got relegated, the 4.2-3.1 was the most prevalent TF with a lower use of the 4.3.3 TF (all *p* < 0.050). In addition, 5.3.2 and 5.4.1 TFs were more prevalent only in teams that got relegated to an inferior category (*p* < 0.050).

Table 2 shows the correlation between the frequency of the TF and the number of points obtained at the end of the season. There was a positive correlation between the use of the 4.3.3 TF and points obtained at the end of the season (*p* < 0.01). However, there was a significant negative correlation between the use of 5.4.1 (*p* < 0.001), 5.3.2 (*p* < 0.001), 4.5.1 (*p* < 0.01) and 4.2-3.1 (*p* < 0.001) TFs.

**Table 2:** Correlation coefficients between the frequency of each team formation and the points obtained at the end of the season.

	3.4.3	3.5.2	4.2-3.1	4.3.3	4.4.2	4.5.1	5.3.2	5.4.1
**Points**	0.078	0.045	0.276	0.214	0.040	0.207	0.353	0.285
***p* value**	0.298	0.549	< 0.001	0.004	0.591	0.005	< 0.001	< 0.001

**Figure 4:**
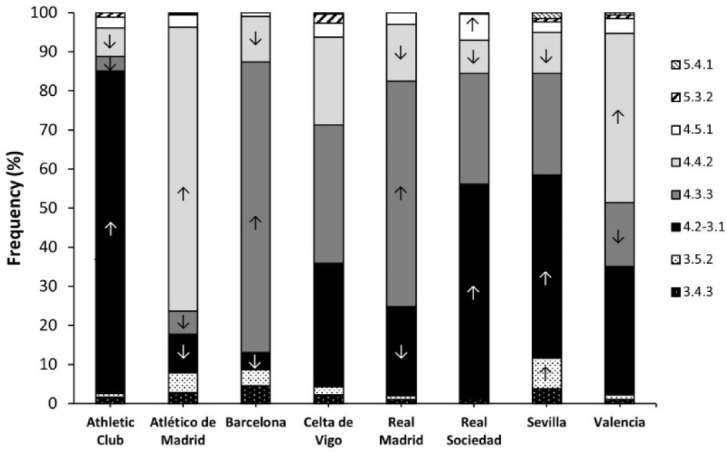
Distribution of teams’ formations in the nine teams that participated in all seasons of LaLiga from 2012/13 to 2020/21. Data are frequencies for each tactic formation within each team. ↑ indicates that the selection of the tactic formation was higher than expected in the team at p < 0.05. ↓ indicates that the selection of the tactic formation was lower than expected in the team (p < 0.05).

Figure 4 shows the distribution of TFs in the nine teams that participated in all the seasons of *LaLiga* included in the study. There was a statistically significant difference in the distribution of TFs implemented by soccer teams (*p* < 0.001).

Overall, the 4.2-3.1 was the most common TF for Athletic Club, Real Sociedad, and Sevilla (*p* < 0.050), the 4.3.3 for FC Barcelona, and Real Madrid CF (*p* < 0.050), and the 4.4.2 for Atlético de Madrid and Valencia CF. Considering RC Celta de Vigo, the most frequent formation was the 4.3.3. None of the teams had a predominance of TFs with three or five defenders.

## Discussion

The aim of this paper was to explore the evolution of TFs during nine seasons of the Spanish *LaLiga*, considering the influence of the team ranking. Our study found a significant change in the use of each TF across all the seasons under investigation. Also, our results showed that the frequency of TFs was affected by the teams’ ranking at the end of the season.

Regarding the evolution of TFs, the 4.2-3.1 TF was the most common tactical structure from 2012/13 to 2015/17, while the 4.4.2 TF was most frequent from 2017/18 to 2020/2021. It was interesting to observe that TFs such as the 4.3.3 and the 4.5.1 showed a relatively stable trend over the seasons, whereas 3.5.2 and 3.4.3 TFs have slightly increased in the last seasons. In relation to this fact, TFs with four defenders were most predominant in all seasons, although a slight change has been found since 2015/16, where the use of three defenders has increased. Additionally, TFs with five midfielders and one forward showed a descending trend, whereas tactical structures with four midfielders and two forwards experienced an upward trend. These findings highlight that the Spanish *LaLiga* is evolving from the use of very few TFs towards a more diverse scenario where teams use a greater variety of TFs.

Our results agree with the analysis of Mesoudi (2020) which observed that the 4.2-3.1 was the most frequent TF in the top five European leagues, followed by 4.3.3 and 4.4.2 TFs. In line with our findings, this study also observed a descending trend of the 4.2-3.1 TF over the years, while the 4-4-2 TF and especially the 4.3.3 TF increased. The study of Guedea-Delgado et al. (2019) also observed that the 4.2-3.1 was the most prevalent TF during the 2018 World Cup, followed by 4.4.2, 4.3.3, 3.5.2, 4.5.1, 5.3.2 and 5.4.1 formations.

The high prevalence of the 4-2-3-1 TF may be due to its appropriate offensive versus defensive balance (Mercé et al., 2017), characterized by a rational distribution of players on the field and great versatility to move and switch positions to attack. In this sense, the four defenders and two midfielders secure an appropriate defensive coverage, allowing the four attacking players to focus more on interacting offensively. Also, this TF offers great tactical versatility, so that small changes in players’ positions can help teams adapt to different match scenarios and modify their positional structure. For example, the 4.2-3.1 TF can change momentarily to a 4.4.1-1, 4.4.2 or 4.5.1 TF in different defensive or offensive moments. Despite its balanced condition, this TF has shown a clear decline over the years, especially in favor of the 4.4.2 TF. A possible reason for this shift could be due to the tendency of coaches to be more conservative defensively. In fact, the study of Errekagorri et al. (2022) suggested that the trend in the evolution of the game in the Spanish *LaLiga* in the last years is that the defensive game prevails over the attacking game. In this way, the 4.4.2 TF increases the defensive protection of teams in comparison to the 4.2-3.1 TF, so that wingers have a more defensive role in the 4.4.2 TF, where they need to join the other two midfielders to form a line of four players, what secures a higher defensive protection. This spatial organization can also make a more efficient use of physical demands of the teams according to the study of Forcher et al. (2022), where they found that the 4.4.2 was the TF with lower physical demands, in comparison with the other TFs. In this sense, the 4.4.2 can be considered the most symmetrical TF, and it might be easier for players to be compact and close to their teammates. In this context, physical demands to protect and cover space would be lower than in other TFs (Forcher et al., 2022). In this line, a recent study by Arjol-Serrano et al. (2021) observed that forwards and central midfielders registered lower physical demands playing in a 4.4.2 than in a 4.2-3.1 TF. This fact may be due to the necessity of covering less space of these positions, so that players’ distances between the midfielders’ line and the forwards’ line in the 4.4.2 TF are potentially lesser. Besides, those authors found that the 4.4.2 TF had a better adaptation to teams with possession-based play styles than the 4.2-3.1 TF, what suggests that the 4.4.2 TF has a balanced positional structure both for defending and for attacking.

Nevertheless, our findings show that it is necessary to consider the team´s ranking to analyze the utilization of TFs in Spanish competitions. According to the ranking, the 4.3.3 is the most frequent TF applied by the champion of the league and is also often used by teams that qualify for the Champions League, after the 4-4-2. In fact, it was the only TF that showed a positive correlation with total points obtained at the end of the season. For the rest of the ranking positions, the 4.2-3.1 was the most frequent TF, followed by the 4.4.2.

The higher prevalence of the 4.3.3 TF in the top tier teams may be due to the greater technical and tactical skills of their players. This fact would allow coaches to implement a more offensive style of play in comparison to the rest of the teams, which would use more “balanced” TFs, such as the 4-2-3-1, 4-4-2 or 4-5-1. In this line, existing literature has shown that high ranked teams register more ball possession (Bradley et al., 2014), higher probabilities to implement a combinative attack rather than a direct attack (González-Rodenas et al., 2021), and greater production of offensive events than medium- and low-ranked teams in different competitions (Brito de Souza et al., 2019; Brito Souza et al., 2019; Gómez et al., 2018; Yang et al., 2018). In this context, the spatial structure of the 4-3-3 TF presents three forwards that should occupy high and wide areas of the field. Additionally, this TF includes two offensive midfielders that ideally should receive the ball behind the line of midfielders of the opposing team. Thus, this positional structure shows a clear predisposition to attack by having the ball possession and progressing by means of short passes, needing skillful players with a high degree of passing accuracy. According to this rationale, our results show that some of the best teams in the world such as Real Madrid CF and FC Barcelona were teams with a higher use of the 4-3-3 TF over the past seasons. According to this rationale, Forcher et al. (2022) observed that all playing positions except for forwards displayed more short passes, middle passes and short possessions in the 4.3.3 and 4.4.2 diamond TFs, compared to the rest of the TFs.

On the other hand, it is interesting to highlight that TFs with five defenders such as the 5-4-1 and 5-3-2 were most used by teams in the middle or relegation positions. This finding is probably due to the urgent necessity of lower ranking teams to concede the least number of goals as possible, to maximize their possibilities to win matches by scoring few goals. This would make coaches implement more conservative TFs (Low et al., 2021) and prioritize the defensive balance and security by accumulating more players in the defensive line.

This research presents several limitations to be considered. Firstly, our data only collected the initial TFs, which registered the primary match strategy chosen by coaches, but excluded the possible modifications in the TF made during the game. Secondly, the exclusive analysis of TFs does not capture the dynamic nature of the game of soccer, where players interact to create emergent behaviors that may lead to multiple positional structures and tactical patterns (Amatria et al., 2019) during different game moments.

### Practical Implications

This study has important practical implications for soccer coaches, sporting directors and tactical analysts. On the one hand, our findings capture the main TFs used in Spanish professional soccer, what provides a comprehensive picture on this topic that lacked strong scientific evidence (Forcher et al., 2022). On the other hand, our results show the evolution and contemporary trends of TFs used by professional teams, what allows soccer coaches and practitioners to reflect on the evolution and possible future direction of soccer tactics.

## Conclusions

In conclusion, our study found a significant change in the use of TFs across all the seasons under investigation. These findings highlight that the Spanish *LaLiga* evolved from the use of very few TFs led especially by the 4.2-3.1 TF towards a more diverse scenario where teams use a greater variety of TFs. In this line, TFs with one forward and five midfielders experienced a descending trend, while TFs with four defenders showed a stable trend throughout the years. Also, our results showed that the implementation of the TF depended on the teams’ ranking at the end of the season. In this sense, the 4.3.3 was the most used TF in the team that won the league, and its frequency was very high in teams that qualified for the UEFA Champions League. In contrast, the 4.2-3.1 followed by the 4.4.2 were the most prevalent TFs for the rest of the ranking groups.
